# Less-invasive subdural electrocorticography for investigation of spreading depolarizations in patients with subarachnoid hemorrhage

**DOI:** 10.3389/fneur.2022.1091987

**Published:** 2023-01-05

**Authors:** Franziska Meinert, Coline L. Lemâle, Sebastian Major, Simeon O. A. Helgers, Patrick Dömer, Rik Mencke, Martin N. Bergold, Jens P. Dreier, Nils Hecht, Johannes Woitzik

**Affiliations:** ^1^Department of Neurosurgery, Carl von Ossietzky University Oldenburg, Oldenburg, Germany; ^2^Research Center Neurosensory Science, Carl von Ossietzky University Oldenburg, Oldenburg, Germany; ^3^Center for Stroke Research Berlin, Charité-Universitätsmedizin Berlin, Corporate Member of Freie Universität Berlin, Humboldt-Universität zu Berlin, Berlin Institute of Health, Berlin, Germany; ^4^Department of Neurology, Charité-Universitätsmedizin Berlin, Corporate Member of Freie Universität Berlin, Humboldt-Universität zu Berlin, and Berlin Institute of Health, Berlin, Germany; ^5^Department of Experimental Neurology, Charité-Universitätsmedizin Berlin, Corporate Member of Freie Universität Berlin, Humboldt-Universität zu Berlin, Berlin Institute of Health, Berlin, Germany; ^6^Department of Anaesthesiology and Intensive Care Medicine, Carl von Ossietzky University Oldenburg, Oldenburg, Germany; ^7^Bernstein Center for Computational Neuroscience Berlin, Berlin, Germany; ^8^Einstein Center for Neurosciences Berlin, Berlin, Germany; ^9^Department of Neurosurgery, Charité-Universitätsmedizin Berlin, Corporate Member of Freie Universität Berlin, Humboldt-Universität zu Berlin, Berlin Institute of Health, Berlin, Germany

**Keywords:** electrocorticography, spreading depolarization, subarachnoid hemorrhage, neurocritical care, Spencer-type electrode array

## Abstract

**Introduction:**

Wyler-strip electrodes for subdural electrocorticography (ECoG) are the gold standard for continuous bed-side monitoring of pathological cortical network events, such as spreading depolarizations (SD) and electrographic seizures. Recently, SD associated parameters were shown to be (1) a marker of early brain damage after aneurysmal subarachnoid hemorrhage (aSAH), (2) the strongest real-time predictor of delayed cerebral ischemia currently known, and (3) the second strongest predictor of patient outcome at 7 months. The strongest predictor of patient outcome at 7 months was focal brain damage segmented on neuroimaging 2 weeks after the initial hemorrhage, whereas the initial focal brain damage was inferior to the SD variables as a predictor for patient outcome. However, the implantation of Wyler-strip electrodes typically requires either a craniotomy or an enlarged burr hole. Neuromonitoring *via* an enlarged burr hole has been performed in only about 10% of the total patients monitored.

**Methods:**

In the present pilot study, we investigated the feasibility of ECoG monitoring *via* a less invasive burrhole approach using a Spencer-type electrode array, which was implanted subdurally rather than in the depth of the parenchyma. Seven aSAH patients requiring extraventricular drainage (EVD) were included. For electrode placement, the burr hole over which the EVD was simultaneously placed, was used in all cases. After electrode implantation, continuous, direct current (DC)/alternating current (AC)-ECoG monitoring was performed at bedside in our Neurointensive Care unit. ECoGs were analyzed following the recommendations of the Co-Operative Studies on Brain Injury Depolarizations (COSBID).

**Results:**

Subdural Spencer-type electrode arrays permitted high-quality ECoG recording. During a cumulative monitoring period of 1,194.5 hours and a median monitoring period of 201.3 (interquartile range: 126.1–209.4) hours per patient, 84 SDs were identified. Numbers of SDs, isoelectric SDs and clustered SDs per recording day, and peak total SD-induced depression duration of a recording day were not significantly different from the previously reported results of the prospective, observational, multicenter, cohort, diagnostic phase III trial, DISCHARGE-1. No adverse events related to electrode implantation were noted.

**Discussion:**

In conclusion, our findings support the safety and feasibility of less-invasive subdural electrode implantation for reliable SD-monitoring.

## 1. Introduction

In recent years, multimodal neuromonitoring has become an important technique in neurocritical care, because immediate online detection of clinical deterioration plays a key role in the prevention of secondary neurological injury (1). This is particularly relevant in neurosurgical patients at risk of delayed cerebral ischemia after aneurysmal subarachnoid hemorrhage (aSAH) since the time-period between the onset of an insult and manifestation of permanent damage in the brain is brief and the consequences of permanent injury are devastating (2).

The dilemma especially in unconscious patients is that the integrity of brain structure and function are less accessible to point-of-care diagnostics (3, 4). While signs of secondary injury can be reliably detected *via* repeated neurological examinations in fully conscious patients, the assessment of secondary neurological injury in patients with reduced consciousness is very limited (4, 5). As a result, treatment is often nonspecific and does not occur at the proper time of injury development, even if appropriate interventions were available. In particular, there is a risk that patients are overtreated, which can worsen the outcome as much as undertreatment. It is also extremely difficult to conduct proof-of-concept treatment trials in this situation. So, it is not surprising that none of the widely used so-called rescue therapies of delayed cerebral ischemia after aSAH have been proven efficacious in randomized trials. Accordingly, their use varies greatly between countries and centers and they might be ineffective or even detrimental (6).

In fact, despite numerous randomized trials, prophylaxis with oral nimodipine remains the only pharmacological therapy that has been shown to reduce the risk of delayed cerebral ischemia and adverse outcome after aSAH (6). Nimodipine prevents one of three poor outcomes due to delayed cerebral ischemia (7), although it has no measurable effect on angiographic vasospasm (8–10). On the other hand, nearly all patients in the recent prospective, observational, multicenter, cohort, diagnostic phase III trial of severe aSAH, DISCHARGE-1, received nimodipine. Nevertheless, 90 of 170 (53%) patients in this MRI-based study developed delayed infarcts despite nimodipine prophylaxis (11). The early focal brain damage volume due to intracerebral hemorrhage and early infarcts was 46 ± 73 ml (56%) and the focal brain damage volume due to delayed cerebral ischemia was 36 ± 80 ml (44%). Nimodipine may be helpful after aSAH *via* its microvascular effects (12), but it by no means sufficiently addresses the clinical problem of delayed cerebral ischemia at present.

To achieve real-time detection of newly developing injury, multimodal monitoring comprises different parameters, of which electrocorticography (ECoG) has recently gained increased attention. Importantly, just as diffusion-weighted MRI, two-photon microscopy or serial section electron microscopy allow visualization of neuronal cytotoxic edema, ECoG in the direct current (DC) frequency range also allows visualization of neuronal cytotoxic edema (13–15). The only difference is that neuronal cytotoxic edema is not referred to as neuronal cytotoxic edema in electrocorticographic language but as spreading depolarization (SD), for historical rather than contextual reasons (14). In addition, SD causes spreading depression of the spontaneous brain activity. Unlike SD, spreading depression is not observed in the DC frequency range but in the alternating current (AC)-ECoG frequency range, where it is visible as a rapidly evolving reduction in the amplitudes of spontaneous activity that spreads between adjacent recording sites along with SD (16). Spreading depression results from the depolarization block that SD produces, but usually lasts longer than SD, suggesting that spreading depression is maintained at a later stage by other mechanisms such as the release of adenosine (17).

It has been suggested that SD variables may serve as a real-time mechanistic biomarker for impending parenchyma damage after aSAH (18–20). To this end, the numbers of SDs, isoelectric SDs, clustered SDs and total (cumulative) SD-induced depression durations (TDDD) were determined for each 24-h period of each patient in DISCHARGE-1 following the initial hemorrhage (11). The peak TDDD (PTDDD) was defined for each patient as the maximal TDDD among all 24-h periods. The results of DISCHARGE-1 suggested that a 25-min cutoff for the TDDD in the just-past 24-h period is an appropriate first “alert level” to review the patient's status and initiate targeted management strategies. This recommendation was made although it is still relatively uncertain after 25 min whether the event will be reversible or progress to infarction. In addition, a 60-min cutoff was proposed as an appropriate moment to initiate rescue therapy because it indicates still reversible delayed neurological deficit with 0.71 sensitivity and 0.82 specificity. A 180-min cutoff indicated delayed infarction with a targeted 0.62 sensitivity and 0.83 specificity. Moreover, SD variables, and specifically PTDDD were included in each multiple regression model for longitudinal neuroimaging-proven early, delayed, and total brain damage, outcome at 7 months, and patient death (11). Interestingly, the median Glasgow Coma Score was the second most powerful neuromonitoring parameter after SD variables in most models. Longitudinal neuroimage segmentation was performed both manually and semi-automatically.

The hallmark of SD is the near-complete breakdown of the transmembrane ionic gradients, although the name, spreading depolarization, refers to the almost complete loss of neuronal membrane potential (16). SDs occur more or less abundantly in many clinical contexts such as migraine aura, transitory ischemic attacks, stroke, brain trauma, brain death development and dying of the brain from cardiac arrest (11, 14, 21–23). The continuum of SDs describes the spectrum from transient events with negative DC shifts of intermediate to short duration in less ischemic or adequately supplied tissue to terminal events in severely ischemic tissue characterized by long lasting DC shifts and transition of the neurons from the state of injury to cell death (19, 24, 25). In normal tissue, SDs cause vasodilation and hyperperfusion to provide sufficient energy for restoration of the near-collapsed transmembrane ionic gradients by energy-dependent membrane pumps. However, in pathological situations, neurovascular coupling may be disrupted, and SD may lead to an inverse hemodynamic response with severe vasoconstriction and spreading ischemia, resulting in a severe mismatch between energy supply and demand, delaying energy-dependent recovery from SD and inducing cell death (12, 26). Using neuromonitoring technology in combination with longitudinal neuroimaging, the entire sequence of both early and delayed brain infarct development after aSAH with SD-induced persistent activity depression, SD-induced spreading ischemia, and the transition of clustered SDs to the negative ultraslow potential (NUP) was demonstrated in a small patient population in which optoelectrodes were directly overlying newly developing infarcts (27). Similar to animal experiments (28, 29), dying of brain tissue is thus indicated by the transition from SD to a NUP in ECoG and to a persistent diffusion restriction in MRI, whereas SD is initially a reversible event.

**Figure 1:**
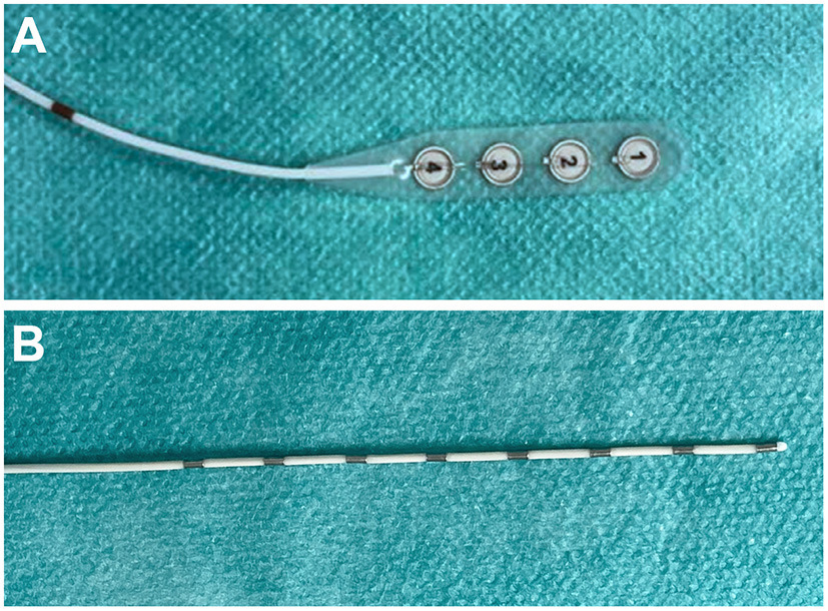
Design of Wyler- and Spencer-type electrode arrays. **(A)** 4-contact Wyler-electrode. Electrode contacts are isolate on the backside of the electrode and have a diameter of 5 mm. **(B)** 8-contact Spencer-type electrode arrays. Contacts are located at the darker part of the electrode with 10 mm intercontact distance and 1.1 mm diameter. In contrast to the Wyler-electrode the Spencer-type electrode arrays have the same diameter across the whole length of the electrode.

For the detection of SDs, so far, subdurally placed Wyler-strip electrodes are considered to be the gold standard electrode technology (Figure 1A) (16, 22, 25, 30). Wyler-strip electrodes can either be placed through a craniotomy or through an enlarged burr hole [compare figure 7 in (26)]. In DISCHARGE-1, Wyler-strip electrodes were placed in 19 of 180 (10.6%) patients through a burr hole. However, this requires an expansion of the burr hole. In order to make ECoG monitoring more widely applicable to patients, we recently reported a less-invasive ECoG electrode implantation approach using subdurally placed Spencer-type electrode arrays (Figure 1B) (31). Due to their conical design, these electrodes can be subdurally implanted through a smaller burr-hole and easily removed at bedside. In the present study, we now investigated whether the ECoG recording quality using this technique is sufficient for reliable neuromonitoring of SDs and SD-induced spreading depression.

## 2. Methods

### 2.1. Study design

We included patients aged 18 years or older (Table 1) that suffered from a severe aSAH and underwent ECoG monitoring for SDs *via* subdural Spencer-type electrode arrays, placed during implantation of an external ventricular drain (EVD). Inclusion criteria were according to the DISCHARGE-1 trial, except for the use of Spencer-type electrode arrays. Informed consent was obtained from all patients or their legally authorized representatives.

**Table 1:** Patient characteristics.

**Patient**	**Age**	**Sex**	**GCS**	**M. Fisher grade**	**Hunt & Hess grade**	**WFNS**	**Aneurysm localization**	**eGOS**
1	58	M	13	3	I	II	MCA	8
2	56	M	13	4	III	II	AComA	6
3	25	W	7	4	IV	IV	AComA	4
4	72	W	3	4	IV	V	MCA	3
5	56	M	13	4	IV	III	AComA	6
6	68	W	4	4	IV	V	Basilar A.	3
7	74	W	14	4	II	II	MCA, AComA	8

Age, age at the insult; GCS, Glasgow Coma Scale at the time point of the first contact; eGOS, extended Glasgow Outcome Scale score at 3–6 months; AComA, anterior communicating artery; Basilar A., basilar artery; MCA, middle cerebral artery; WFNS, World Federation of Neurosurgical Societies Score.

### 2.2. Subdural electrocorticography

ECoG electrode implantation in patients suffering aSAH was performed as previously described (31) after securing of the responsible aneurysm either by microsurgical clipping or endovascular therapy. Briefly, a Spencer-type electrode array (6, 8 or 12 platinum contacts with 10 mm distance between contacts, 1.1 mm diameter, AD-Tech Medical Instrument Corporation, Oak Creek, Wisconsin, USA) was subdurally placed on the cortex *via* a burr hole for implantation of an EVD. The electrode was oriented transversely to a viable cortical region that belonged to the vascular territory of the aneurysm-carrying vessel, because this cortex is often covered with blood and thus is a predilection site for delayed cerebral ischemia (16). After EVD and ECoG electrode implantation, patients were transferred to our intensive care unit (ICU) and ECoG monitoring was performed for a period of 5–10 days (Table 2). Once it was clinically determined that ECoG monitoring was no longer needed, the Spencer-type electrode array was removed at the bedside.

**Table 2:** SD characteristics.

**Patient**	**Total number of SDs**	**Number of iSDs**	**Number of clustered SDs**	**PTDDD (min)^a^**	**Total monitoring duration (h)**
1	39	0	34	306.6	201.3
2	9	3	3	87.8	201.6
3	5	0	0	51.6	217.2
4	0	0	0	0	194.0
5	1	0	0	39.3	6.7
6	26	1	10	181.5	315.5
7	4	0	1	75.6	58.1

a Peak total SD-induced depression duration (PTDDD) of a recording day in minutes.

### 2.3. Patient management

On the ICU, the electrode was connected to a Moberg Neuromonitoring system (Moberg CNS, Moberg Research, Inc., Ambler, Pennsylvania, USA) for online bedside ECoG recording and monitoring. Intracranial pressure (ICP) was continuously monitored, and patients remained intubated and sedated until ICP was within normal ranges. Additional bedside neuromonitoring included brain tissue partial tissue pressure of oxygen (ptiO_2_) in selected cases (Licox, Integra Lifesciences Corporation, Plainsboro, NJ, USA). A critical ICP threshold was defined as ICP >20 mmHg for longer than 10 min and treated with cerebrospinal fluid drainage, osmotic therapy using mannitol (125–250 ml, maximum every 4 h) and/or hypertonic saline after controlling the blood electrolytes, and/or deep sedation. A routine postoperative computerized tomography scan (CT) was performed within 24 h to rule out procedure-related complications. Treatment was performed according to the guidelines of the German Society of Neurosurgery.

### 2.4. Data processing

Recording and analysis of ECoG data and interpretation of SDs was performed following the recommendations of the COSBID study group (11, 16, 19). Briefly, for each patient the following parameters were determined: (i) total number of SDs; (ii) number of SDs in electrically inactive tissue (isoelectric SDs); (iii) number of clustered SDs (i.e., SDs that occurred 1 h apart from the previous SD); (iv) peak total SD-induced depression duration of a recording day (PTDDD).

## 3. Results

### 3.1. Patient characteristics

Overall, we included seven patients (four females, three males; median age: 58 years; interquartile range: 56–72) that fulfilled the inclusion criteria. All patients suffered severe aSAH with a median Hunt and Hess grade 4 (interquartile range: 2–4) and a median modified Fisher Grade 4 (interquartile range: 4–4). At presentation in the hospital patients had a median Glasgow Coma Scale of 13 (interquartile range: 4–13) and a median World Federation of Neurosurgical Societies Score (WFNS) score of 3 (range 2–5). Detailed patient characteristics are presented in Table 1.

### 3.2. ECoG monitoring

**Figure 2:**
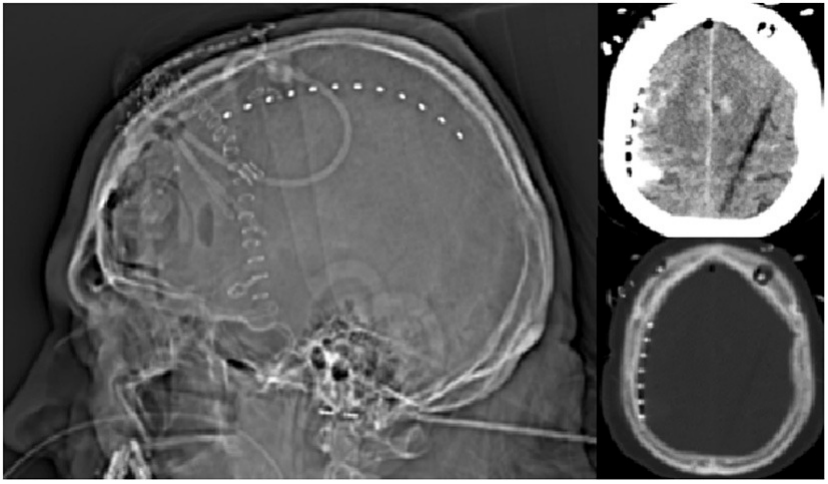
Electrode localization on imaging. Localization of the Spencer-type depth electrode (12 contact electrode) after implantation *via* a burr hole.

**Figure 3:**
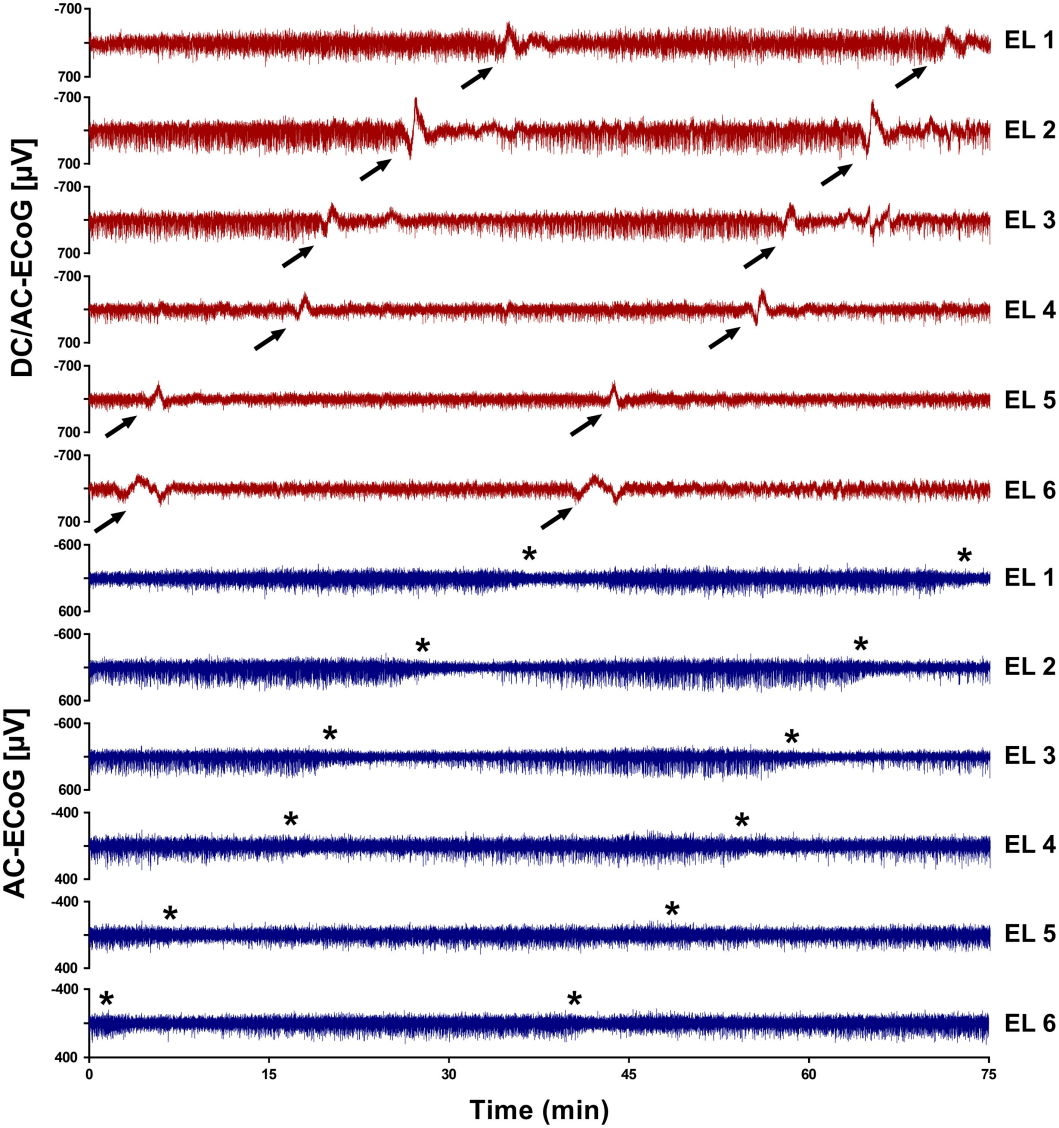
Subdural ECoG recording traces of Spencer-type electrodes arrays. In this case (patient No. 1), monitoring was performed using a 6-contact Spencer-type electrode array placed through a right frontal burr-hole. The occurrence of clustered SDs can be observed by the large negative shift in the direct current ECoG (DC/AC-ECoG; band-pass: 0.01–45 Hz; red traces, arrows). The SDs induced depression of the spontaneous cortical activity in the alternating current range (AC-ECoG; band-pass: 0.5–45 Hz, blue traces, asterisks show the beginning of a depression period). EL, electrode.

**Figure 4:**
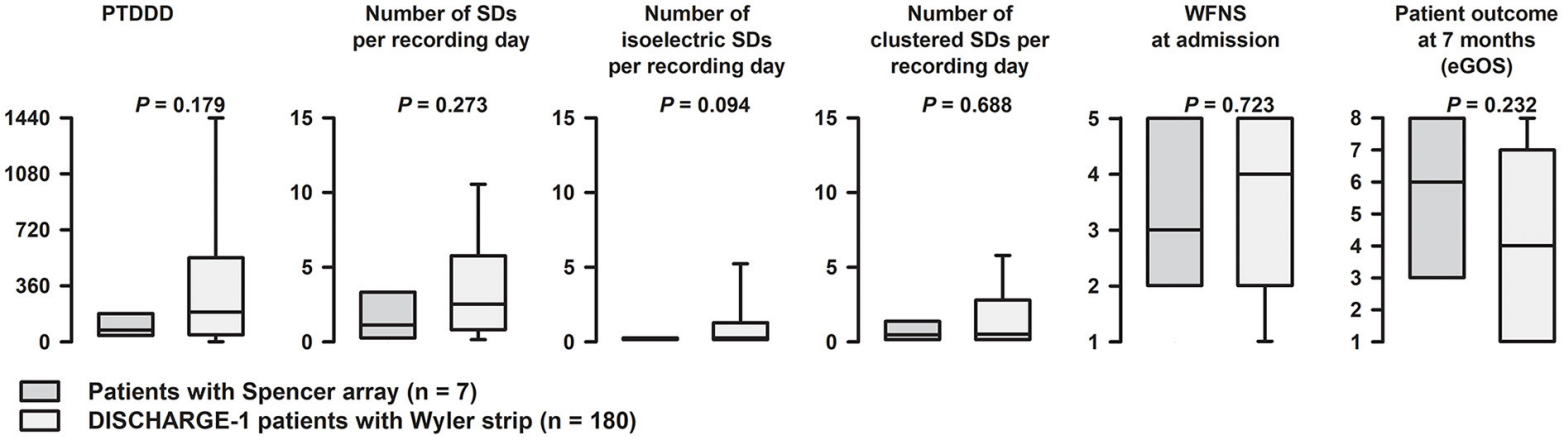
Comparison of the monitoring quality of Spencer-type electrode arrays and Wyler-strips. In this figure the four different SD variables were compared between Spencer-type electrode arrays (*n* = 7 patients) and Wyler-strips (*n* = 180 patients from the DISCHARGE-1 trial). There was a tendency for patients in the present study to be slightly less affected than patients in DISCHARGE-1, but this concerned not only SD variables but also WFNS at admission and eGOS at 7 months. Importantly, no significant differences were observed. eGOS, extended Glasgow Outcome Scale score at 3–6 months; PTDDD, peak total SD-induced depression duration of a recording day in minutes; WFNS, World Federation of Neurosurgical Societies Score.

Implantation of the Spencer-type electrode array (Figure 2) was successfully performed without complications in all patients. During the ICU monitoring period, no electrode-associated bleeding, infection, or cerebrospinal fluid fistula were noted. Also, no complications were noted during electrode removal at bedside. Overall, a total of 84 SDs were recorded in 6/7 patients during a total monitoring duration of 1,194.5 h, which corresponded to a median monitoring period of 201.3 (interquartile range: 126.1–209.4) hours per patient. Four SDs (4.8%) were isoelectric and 48 (57%) occurred in clusters (Figure 3, Table 2). The median PTDDD was 75.6 (interquartile range: 45.5–134.7) minutes. In one of the seven patients, no SD was detected throughout the entire monitoring period. We compared the PTDDD as well as numbers of SDs, isoelectric SDs and clustered SDs per recording day with the corresponding numbers of the DISCHARGE-1 trial and did not find a significant difference (Figure 4).

### 3.3. Outcome

At 7 month after the initial hemorrhage, the patients had reached a median extended Glasgow Outcome Scale (eGOS) score of 6 (range: 3–8), representative of persisting moderate neurological deficits.

## 4. Discussion

Electrophysiological monitoring of patients requiring neurointensive care has become increasingly important in recent decades (18, 30, 32), as SDs have been shown to serve as real-time biomarkers for the development of secondary brain injury (11). Even though correlates of SDs and SD-induced activity depression have been noted in conventional scalp EEG (33, 34) or EEG with epidural peg electrodes (22), this is currently not sufficient for clinical decision making (16, 35). Thus, the gold-standard for robust SD detection remains subdural ECoG recording performed using platinum/iridium Wyler-strip electrodes (16, 18, 22, 25, 26, 30).

In the present pilot study, we performed subdural ECoG monitoring in seven patients suffering aSAH to determine the feasibility of using less-invasive platinum/iridium Spencer-type electrode array for clinical SD monitoring. We specifically chose aSAH because, along with traumatic brain injury (TBI) (36), it is the best-studied form of severe acute brain injury in which SD monitoring could provide clinical benefit by detecting secondary neurological deterioration in a timely manner and allowing the treating intensivist to provide treatment stratification before infarction has developed (11). To determine the feasibility of Spencer-type electrode arrays for this purpose, recordings of all patients were analyzed regarding the occurrence of SDs, but also regarding the possibility to generate and quantify additional SD parameters that are commonly used for more detailed SD characterization beyond the occurrence of an SD event alone. Most importantly, all recordings provided sufficient data quality in order to be analyzed using the standard methods (16) without requiring additional steps for artifact removal. Further, ECoG recordings in our patients showed similar SD characteristics like those previously described using Wyler-strip electrodes for subdural SD recording in comparable cohorts (18, 37). Notably, the distributions of SD variables within our study were not significantly different from those observed in DISCHARGE-1. There was a tendency for patients in the present study to be slightly less affected than patients in DISCHARGE-1, but this concerned not only SD variables but also WFNS at admission and eGOS at 7 months.

Although our assessment remains inherently limited by the fact that we did not have an additional Wyler-strip inserted next to the Spencer-type electrode array to serve as a direct control, the coherence between our present and previous experience supports the general feasibility of using Spencer-type electrode arrays for robust subdural ECoG and reliable SD detection at bedside in the ICU setting.

In contrast to patients with large craniotomies where subdural electrode placement is considered a straightforward procedure, subdural electrode implantation for ECoG recording is far less common in patients that do not require craniotomy. Although Wyler-strip electrodes can been subdurally inserted *via* enlarged burr holes, for example in epilepsy surgery (38–40) or to record SDs (26) this procedure is more difficult to handle in a routine setting for ICU monitoring. In contrast, the subdural use of smaller Spencer-type electrode arrays could bear several advantages during implantation and removal, considering their thin cylindrical architecture and equal diameter of merely 1.1 mm. During implantation, only a regular burr-hole is required and the direction of electrode insertion into the subdural space can be precisely guided. Wyler-strips can also be removed at the bedside (16), but the removal of Spencer-type electrode arrays is even easier and less complicated because of their small and uniform diameter.

Apart from these advantages regarding handling, data quality of ECoG recordings is of major importance to ensure reliable SD detection. Importantly, data quality relies on the direct contact of the electrode with the surface of the brain. In the case of the conventional Wyler-strip electrodes, this is ensured by their flat and sheet-like contact surface, where electrode contacts are only located on one side, whereas the other side of the Wyler-strip is isolated. In contrast, the contacts of depth electrodes are located around the circumference of the entire electrode and therefore, the Spencer-type electrode array has direct contact not only to the cortical surface but also to the dura mater lying above. Against this background, an important electrophysiological finding was that we did not detect any relevant artifacts. Possibly, this is explained by the fact that the dura itself is an electrically inactive tissue, which isolates the electrode surface opposite from the brain surface. Also, the circumferential electrode contacts provide the additional advantage that Spencer-type electrode arrays do not require implantation according to a specific orientation, since subdural contact to the brain in ensured in every direction.

In conclusion, our study provides the first evidence that the use of Spencer-type electrode arrays for subdural ECoG monitoring for the purpose of SD detection is feasible. The ability to implant the electrode through a small burr hole, together with the advantage of easy and safe bedside removal and high-quality ECoG recording, may help to make this less invasive technology available to patients who do not require craniotomy, representing an important step toward the introduction of routine clinical SD monitoring in the ICU. However, the findings should still be validated in a larger clinical trial. In addition, it should be investigated in swine, for example, how good SD recordings are in direct comparison when recording in parallel with Wyler-strips and Spencer-type electrode arrays.

## Data availability statement

The raw data supporting the conclusions of this article will be made available by the authors, without undue reservation.

## Ethics statement

The studies involving human participants were reviewed and approved by Medizinische Ethikkommission Universität Oldenburg. The patients/participants provided their written informed consent to participate in this study.

## Author contributions

FM and JW contributed substantially to the conception and design of the work, to patient enrollment and monitoring, and drafting and revising the manuscript for important intellectual content. SH, PD, MB, and RM contributed to data collection, analysis, edited, and approved the manuscript. CL, SM, and NH contributed to data analysis, edited, and approved the manuscript. JD contributed to study design, edited, and approved the manuscript. All authors contributed to the article and approved the submitted version.
